# Effects of AM/PM Feeding Regimen on Productive Performance, Egg Quality, Bone Properties, Blood Metabolites and Nutrient Utilization in the Aged Laying Hens

**DOI:** 10.1002/vms3.70754

**Published:** 2026-01-14

**Authors:** Mina Toroghian, Heydar Zarghi, Hassan Kermanshhahi, Ali Javadmanesh

**Affiliations:** ^1^ Department of Animal Science Faculty of Agriculture Ferdowsi University of Mashhad Mashhad Iran

**Keywords:** laying hens, performance, split feeding

## Abstract

**Background:**

The nutrient requirements of laying hens are not static but dynamic, changing throughout the day in response to the cyclic nature of egg formation.

**Objectives:**

The current study aimed to evaluate the impact of the AM/PM feeding regimen (AM/PM‐FR), which involved higher levels of protein, amino acids and available phosphorus, and lower levels of Ca in the morning diet, with the converse in the afternoon diet, on aged laying hens.

**Methods:**

A total of 300, 74‐week‐old Hy‐Line W36 laying hens were randomly assigned to a completely randomized design with 5 treatments, 6 replicates, and 10 hens per replicate. Experimental treatments included offering AM/PM‐FR at 0% (control), 10%, 20%, 30% and 40% changing nutrient levels (CNL) between the morning (5:00 AM–2:00 PM) and evening (2:00 PM–5:00 AM) diets. The exposure program consisted of continuous lighting from 5:00 AM to 9:00 PM and darkness from 9:00 PM to 5:00 AM.

**Results:**

The feed conversion ratio (FCR) and economic profit, income minus feed cost (IMFC), improved with a quadratic trend in response to increased CNL of AM/PM‐FR. Throughout the entire experimental period, birds fed AM/PM‐FR at 30% CNL showed improvements of 4.57% in FCR and 17.55% in IMFC compared to the control group. A lower incidence of egg fractures and a higher yolk crude protein concentration were observed with a linear trend in response to increased CNL of AM/PM‐FR. By increasing CNL of AM/PM‐FR, the total tract apparent mineral (Ca and phosphorus) and ether extract retention improved with quadratic and linear trends, respectively. Non‐significant effects of the treatments were noted on egg quality, blood metabolites, bone mechanical properties and mineral contents.

**Conclusions:**

It was concluded that the use of AM/PM‐FR at 30% CNL in aged laying hens may facilitate the precision nutrition and thus improve production and economic performance.

## Graphical abstract

The current study findings endorse the AM/PM‐FR (feeding a high protein, amino acids and phosphorus and low Ca diet in the morning on the contrary in the afternoon) as a favourable practice for egg producers, providing a cost‐efficient and resource‐effective approach to layer production. Although the AM/PM‐FR strategy requires entailing initial investments for existing and housing setups; nevertheless, growing confidence in advancing equipment and IT solutions supports its adoption.

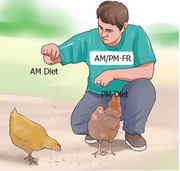

## 1 Introduction

Current laying hens can maintain their production viability into older age, leading to a growing trend among egg producers to keep hens for up to 100 weeks to enhance profitability and sustainable production (Molnár et al. 2016; 2017). However, as an animal ages, its ability to digest and absorb nutrients declines, resulting in a decrease in eggshell quality (Molnár et al. 2016; Roberts and Chousalkar 2013). Therefore, a comprehensive strategy that includes optimal nutrition aimed at improving shell quality, along with various management measures to address health and welfare issues, is essential for successfully extending the egg‐laying cycle (Molnár et al. 2016).

Feeding techniques are vital in the farming of laying hens to ensure that the birds' nutrient requirements are met accurately and efficiently for sustainable production (Jahan et al. 2024). Recent findings suggest that laying hens have varying nutrient needs throughout the day (Molnár, Kempen, et al. 2018a, Molnár, Maertens, et al. 2018b). This hypothesis is based on the understanding that egg components (albumen and shell) are deposited at different times of the day due to the cyclic nature of egg formation. According to egg production physiology, hens require a higher amount of protein and amino acids in the morning when the egg white is deposited compared to the afternoon meal (Hiramoto et al. 1990; Penz and Jensen 1991). Conversely, there is a greater demand for calcium (Ca) for eggshell formation in the afternoon (Kebreab et al. 2009). A new feeding strategy that has recently gained attention among laying hen producers is the practice of providing diets tailored to specific nutritional and physiological needs throughout the day, known as the AM/PM feeding regimen (AM/PM‐FR).

The AM/PM‐FR, also referred to as split feeding, involves providing laying hens with customized diets that address their specific nutritional and physiological needs at different times of the day. This method is based on a fundamental understanding of layer hens' natural feeding behaviour and physiology, which can significantly impact egg production, egg quality and health (Jahan et al. 2024). The AM/PM‐FR is a carefully structured feeding regimen that divides the daily feed allocation into two distinct dietary formulas, typically offered separately in the morning and afternoon, rather than providing a continuous diet throughout the day as seen in traditional feeding systems (Molnár, Kempen, et al. 2018a, Molnár, Maertens, et al. 2018b; Waldroup and Hellwig 2000). The practice of AM/PM‐FR includes offering a diet high in protein, amino acids and phosphorus but low in Ca in the morning, and conversely in the afternoon (Traineau et al. 2015; Van Emous and Mens 2021). This approach could lead to significant cost savings for egg producers (Jahan et al. 2024).

Given that the AM/PM‐FR is a novel nutritional concept, there are limited studies available in this area, and most previous research has focused solely on Ca and phosphorus at a single level of change. The aim of the current study was to investigate the effects of varying levels of nutrients (Ca, phosphorus, protein and amino acids) in the AM/PM‐FR on productive and economic performance, egg quality, bone mechanical properties and mineral content, blood metabolites and nutrient utilization in aged laying hens (76–87 weeks of age).

## 2 Materials and Methods

### 2.1 Birds, Housing and Treatments

The current experiment was conducted at a professional laying farm with a capacity of 90,000 layers in Mashhad, Iran (59°10′44″ E longitude, 36°43′39″ N latitude and an elevation of 1057 m). A total of 300, 74‐week‐old Hy‐Line W36 laying hens were utilized in the experiment. The birds were randomly assigned in a completely randomized design (CRD) with five treatments, six replicates per treatment and 10 birds per replicate. The birds were housed five per cage (50 cm × 45 cm wire‐bottomed cage, corresponding to 450 cm^2^ per hen), with every two adjacent cages containing 10 birds, serving as an experimental unit.

Experimental treatments included changing nutrient levels (CNLs) of AM/PM‐FR, increasing protein, amino acids and available phosphorus while decreasing Ca concentration in the morning diet from 5:00 AM to 1:00 PM, and vice versa in the afternoon diet from 1:00 PM to 9:00 PM at different levels (0% = control treatment, 10%, 20%, 30% and 40%). Experimental diets were formulated based on a least‐cost equation using user‐friendly feed formulation software (UFFDA 1992) to meet the specific dietary requirements outlined by the Hy‐Line breed's recommendations (Hy‐Line 2020), tailored to the hens' age and production levels (Table 1). The exposure program consisted of continuous lighting from 5:00 AM to 9:00 PM and darkness from 9:00 PM to 5:00 AM. The experiment lasted for 12 weeks and was divided into three consecutive periods of 28 days each.

**TABLE 1:** Ingredients and nutrients composition of experimental diets.

	Experimental treatments^a^								
	0%	10%		20%		30%		40%	
Items	AM‐PM diet	AM diet	PM diet	AM diet	PM diet	AM diet	PM diet	AM diet	PM diet
**Ingredients (as fed basis), g/kg**									
Corn	63.63	61.04	66.28	58.44	68.93	55.86	71.6	53.29	74.27
Soybean meal	21.71	25.47	17.86	29.24	14.01	33.00	10.15	36.76	6.30
Soybean oil	1.35	1.29	1.39	1.24	1.42	1.18	1.46	1.12	1.49
DCP	1.29	1.46	1.13	1.62	0.96	1.79	0.80	1.95	0.63
Limestone	11.01	9.71	12.32	8.40	13.63	7.10	14.93	5.79	16.24
Common salt	0.38	0.38	0.38	0.38	0.39	0.37	0.39	0.37	0.39
Vitamin Premix^b^	0.25	0.25	0.25	0.25	0.25	0.25	0.25	0.25	0.25
Mineral Premix^c^	0.25	0.25	0.25	0.25	0.25	0.25	0.25	0.25	0.25
DL‐Methionine	0.13	0.15	0.11	0.18	0.09	0.20	0.07	0.22	0.05
l‐Lysine HCl	0.00	0.00	0.03	0.00	0.06	0.00	0.09	0.00	0.12
l‐Threonine	0.00	0.00	0.00	0.00	0.01	0.00	0.01	0.00	0.01
**Determined nutrients composition** ^d^ **, as‐fed basis**									
ME (kcal/kg)	2800	2800	2800	2800	2800	2800	2800	2800	2800
CP (%)	14.00	15.40	12.60	16.80	11.20	18.20	9.80	19.60	8.40
Ca (%)	4.52	4.07	4.97	3.62	5.42	3.16	5.88	2.71	6.33
Available P (%)	0.36	0.40	0.32	0.43	0.29	0.47	0.25	0.50	0.22
Na (%)	0.17	0.17	0.17	0.17	0.17	0.17	0.17	0.17	0.17
Dig. Lys (%)	0.65	0.72	0.59	0.78	0.52	0.85	0.46	0.91	0.39
Dig. Met (%)	0.34	0.38	0.31	0.42	0.27	0.46	0.24	0.50	0.20
Dig. SAAs (%)	0.55	0.61	0.50	0.66	0.45	0.72	0.39	0.77	0.34
Dig. Thr (%)	0.47	0.52	0.42	0.56	0.38	0.61	0.33	0.66	0.28

Abbreviations: Ca, calcium; CP, crude protein; DCP, Dicalcium phosphate; Dig. Lys, digestible lysine; Dig. Met, digestible methionine; Dig. SAA, digestible Sulphur amino acid; Dig. Thr, digestible threonine; ME, metabolizable energy; Na, sodium; P, phosphorus.

^a^Percentage change of nutrients between the morning and evening diets.

^b^Vitamin premix supplied the following per kilogram of diet; Vitamin A (all‐trans‐retinol), 8000 IU; Vitamin D3 (cholecalciferol), 3300 IU; Vitamin E (α‐tocopherol), 20.0 mg; Vitamin K3 (menadione), 2.5 mg; Vitamin B1 (thiamin), 2.5 mg; vitamin B2 (riboflavin), 5.5 mg; Vitamin B3 (niacin), 30.0 mg; Vitamin B5 (pantothenic acid), 8.0 mg; Vitamin B6 (pyridoxine), 4.0 mg; Vitamin B9 (folic acid), 0.9 mg; Vitamin B12 (cyanocobalamin), 0.023 mg; Vitamin H2 (biotin), 0.075 mg; choline chloride, 487.5 mg, antioxidant 1.0 mg

^c^Mineral premix supplied the following per kilogram of diet; Zn (zinc oxide), 80 mg; Mn (manganese oxide), 90; Fe (iron sulfate), 40; Cu (copper sulphate), 8; I (ethylene diamine dihydroiodide), 1.2; Se (Sodium Selenite), 0.22.

^d^The determined ingredient analysis was used to calculate nutrient composition (crude protein, calcium and sodium were measured by the AOAC (2002) methods; metabolizable energy, digestible amino acids and available phosphorus were measured by NIR analysis.

### 2.2 Production Performance

Egg production (number and weight) and mortality were recorded daily, while performance traits were calculated and compiled at 28‐day intervals (76–79, 80–83 and 84–87 weeks of age). During the experimental periods, the following productive performance traits were measured: feed intake (FI), egg production (EP), average egg weight (EW), egg mass (EM) and feed conversion ratio (FCR). FI was determined by subtracting the amount of feed remaining at the end of each period from the total feed provided during that period, adjusted for mortality. The daily absolute consumption of metabolizable energy (ME), crude protein (CP), Ca, available phosphorus and digestible amino acids (DAAs) such as Lys, Met, Met + Cys and Thr was calculated based on FI and the analysed experimental diet composition. The FCR was calculated as FI divided by EM. Economic profit was assessed as egg income minus feed cost (IMFC).

### 2.3 Egg Quality Traits

At the end of each period, four eggs from each replicate (24/treatment) were randomly selected and transported to the Egg Quality Lab within 6 h of collection for egg quality evaluation. The sampled eggs were weighed using a digital electronic scale (0.001 g, Model GF 400; A&D Weighing). Additionally, the eggs were weighed while submerged in distilled water to calculate the egg specific gravity (ESG) using the Archimedes method, according to the following formula (Hempe et al. 1988).

$$\mathrm{Egg}\;\mathrm{specific}\;\mathrm{gravity},\;g.\;cm^{-3}=\frac{\mathrm{Egg}\;\mathrm{weight}}{\left(\mathrm{Egg}\;\mathrm{weight}\;-\;\mathrm{Egg}\;\mathrm{weight}\;\mathrm{immersed}\;\mathrm{in}\;\mathrm{water}\right)}$$

The egg was then carefully broken onto a glass plate (35 × 25 cm) to evaluate internal quality characteristics. The Haugh unit (HU) was calculated based on the following formula (Eisen et al. 1962):

$$\begin{array}{ccc} & & \mathrm{Haugh}\;\mathrm{unit}\;\\ & & \;=\;100\times \log[\mathrm{Albumen}\;\mathrm{height}\;\left(\mathrm{mm}\right)+7.57-{\left(1.7\times \mathrm{Egg}\;\mathrm{weight}\;\left(g\right)\right)}^{0.37}] \end{array}$$

The height of the structural thick white was measured at the proximal end of the line of least curvature on the section of the structural thick white with the largest uninterrupted surface area. The height measurement was divided by the mean width, and the resulting fraction was termed the albumen index (Heiman and Carver 1936). Yolk and albumen were separated using a commercially available handheld egg separator. A damp cloth napkin was employed to remove any residual albumen from the yolk, which was then weighed. The eggshell was rinsed with water, dried for 48 h, and weighed. Eggshell thickness (ST) was measured using a micrometre (0.001 mm, Model 293‐240; Mitutoyo) at three different locations (air sack, equator and sharp end), and the average was calculated to determine the eggshell thickness. The albumen weight was computed by subtracting the combined weight of the yolk and shell from the total EW (Omary et al. 2024).

### 2.4 Egg Composition

At the end of the experiment, four eggs from each replicate were randomly selected for analysis of egg composition. After breaking, the egg components, including yolk and albumen, were separated as described. Yolks and albumen were pooled into separate containers and homogenized to create composite samples. The solid content of yolk and albumen was determined by drying them in an electric oven at 75°C for 72 h (930.15). The ether extract (EE) content of yolk was measured using the extraction method (920.39). The nitrogen content of yolk and albumen was analysed with a nitrogen analyser (984.13) as described by the Association of Official Analytical Chemists (AOAC 2002).

### 2.5 Blood Collection and Analysis

At the end of the experiment, at 12 o'clock, one bird from each replicate (six/treatment) was randomly selected and a blood sample was collected from the brachial vein of each bird into non‐heparin tubes. Blood samples were centrifuged at 1900 × *g* for 5 min at 4°C to extract serum (Hossaninejad et al. 2021). Serum samples were separated and the blood metabolites (Ca, phosphorus, uric acid, creatinine, total protein, albumin) and liver functional enzyme (alanine aminotransferase, aspartate aminotransferase and alkaline phosphatase) measured using a multi‐test automated random‐access system auto‐analyser (Cobas Bio, Roche, Basel, Switzerland) with kits from Pars Azmoon Company, Iran.

### 2.6 Apparent Nutrient Retention

At the end week of experiment (87 weeks of age), one bird per each replicate (six/treatment) randomly was selected and placed in an individual metabolic cage (40 × 40 cm) and fed their respective experimental diets for 1 week. Total tract apparent nutrient's retention (TTANR) was measured by the total collection method. After a 3‐day adaptation period, excreta samples were collected daily from each cage for four consecutive days, frozen and stored in plastic bags (−20°C) until analysis (Gracia et al. 2003). Prior to analysis, excreta samples were thawed overnight, homogenized, dried (72 h; 60°C). The feed and dried excreta samples were ground (1‐mm screen) and analysed as described chemical analysis section. The TTANR coefficients were calculated for dry matter (DM), EE, CP, ash, Ca and phosphorus according to the following formula (Prola et al. 2013).

$$\mathrm{TTANR},\;\%\;=\frac{\left(\mathrm{Total}\;\mathrm{nutrient}\;\mathrm{ingested}\;\right)-\left(\mathrm{total}\;\mathrm{nutrient}\;\;\mathrm{excreted}\right)}{\left(\mathrm{Total}\;\mathrm{nutrient}\;\mathrm{ingested}\right)}\;\;\times 100$$

### 2.7 Tibia Bone Mechanical Properties and Mineral Contents

At the last week of the experiment, one hen from each experimental unit (six birds/treatment) was randomly selected, weighed and euthanized via cervical dislocation. Immediately after slaughter, each tibia bone was cut, and any adhering tissue was scraped off. The bones were then placed in a sealed plastic bag and stored at −20°C for further examination. In the subsequent analysis steps, the right tibia bone underwent optometry measurements and strength tests, while the left tibia bone was reserved for mineral content analysis.

After thawing overnight at the room temperature, the length and width of the right tibia bone were measured using a digital caliper (0.05 mm, Model 1116–150, Insize Co. Ltd., Suzhou, China), followed by weighing it on a digital electronic scale (0.001 × *g*, Model GF 400, A&D Weighing Co. Ltd., CA.). The weight of each bone was determined when submerged in distilled water to calculate specific gravity (SG) using the Archimedes method and the following formula (Muhammad et al. 2013):

$$\;\mathrm{Bone}\;\mathrm{specific}\;\mathrm{gravity},\;g.cm^{-3}=\frac{\mathrm{Bone}\;\mathrm{weight}}{\left(\mathrm{Bone}\;\mathrm{wight}-\mathrm{Bone}\;\mathrm{weight}\;\mathrm{immersed}\;\mathrm{in}\;\mathrm{water}\right)}$$

Using the three‐point bending test method, the mechanical properties of bones were determined using an Instron Universal Testing Machine (Model H5KS, Tinius Olsen Company). The support span was set at 40% of the bone length (Muszyński et al. 2018). The bone was loaded in the anterior–posterior plane, and a perpendicular load cell was utilized to apply loading to the midpoint at a displacement rate of 5 mm per minute until fracture (Cufadar et al. 2011). The results were recorded as: the force (N) required to achieve structural failure of the tibia, stiffness (N/mm) and energy (J) for each bone.

The left tibia bones were removed from the freezer and placed on a countertop to thaw. Subsequently, they were weighed and dried at 55°C for 72 h in a forced‐ventilation oven. Afterward, the samples were extracted in a Soxhlet extractor for 8 h, then returned to the forced‐ventilation oven at 55°C for another 72 h, and weighed again to determine the DM (Xavier et al. 2015).

### 2.8 Chemical Analysis

The feed, excreta and bone samples were ground (1‐mm screen) and mixed thoroughly before analysis. The samples were chemically analysed for moisture by oven drying method (930.15), ash by muffle furnace (942.05), CP by Kjeldahl method (984.13) and EE by Soxhlet fat analysis (920.39) as described by the Association of Official Analytical Chemists (AOAC 2002) and for mineral concentration (Ca, phosphorus, Mg, Mn and Zn) using an inductively coupled plasma optical emission spectrometer (ICP‐OES; Agilent Australia, Victoria, Australia). After adding 0.1 g of weighted ash to 5 mL of concentrated (65%) nitric acid, the samples were predigested for 100 min at 80°C in a bain‐marie. Following digestion, the digested solution was combined with 20 mL of high‐purity deionized water, and the solution was quantitatively filtered through filter paper, transferred to a 50 mL container, and adjusted to volume with high‐purity deionized water (Fleischer et al. 2014). Analysis for Ca, phosphorus, Mg, Mn and Zn was performed using an ICP‐OES (Agilent Australia, Victoria, Australia).

### 2.9 Statistical Analysis

All data were analysed for normality using SAS 9.1 software through the Univariate plot normal procedure. Subsequently, data were examined using the statistical software SAS 9.4 General Linear Model procedure (SAS 2014). Means were compared using the Tukey test when the effects of dietary treatments were significant at the 5% level (Tukey 1991). Orthogonal polynomials for linear and quadratic responses to dietary treatment were calculated to investigate the relationships between the CNL of AM/PM‐FR as independent variables and the respective traits as dependent variables.

## 3 Results

### 3.1 Feed, Energy and Nutrients Consumption

The effects of CNL of AM/PM‐FR on daily feed, ME, CP, Ca, available phosphorus and DAAs consumption is presented in Table 2. The average daily consumption of feed, ME, CP, available phosphorus and DAAs during the first period (76–79 weeks of age) decreased (linear trend, *p* < 0.05) with increasing CNL of AM/PM‐FR. However, during the second period (80–83 weeks of age), third period (84–87 weeks of age) and throughout the experimental period (Weeks 76–87) these traits were not significantly affected by the experimental treatments.

**TABLE 2:** Effect of AM/PM‐FR on calculated daily absolute intakes of energy and selected nutrients in the aged laying hens.^1^

Items	AM FI	PM FI	Daily FI	AM/PM ratio	ME	CP	Ca	Ava. P	Dig Lys	Dig Met	Dig SAAs	Dig Thr
	g/h/d				kcal/h/d	g/h/d		mg/h/d				
Treatments^2^		76–79 weeks of age										
0%	39.38^a^	52.81^ab^	92.18^ab^	74.57	258^ab^	12.91^a^	4.17	332^a^	599^a^	313^ab^	507^ab^	433^a^
10%	40.04^a^	54.84^a^	94.88^a^	73.01	266^a^	13.08^a^	4.35	336^a^	612^a^	322^a^	518^a^	439^a^
20%	38.65^ab^	49.13^b^	87.78^ab^	78.67	244^ab^	11.88^b^	4.04	306^b^	552^b^	292^bc^	472^bc^	399^b^
30%	38.48^ab^	53.29^ab^	91.77^ab^	72.21	257^ab^	12.23^ab^	4.35	314^ab^	572^ab^	305^ab^	485^ab^	411^ab^
40%	36.98^b^	49.32^b^	86.30^b^	74.98	242^b^	11.39^b^	4.12	293^b^	529^b^	284^c^	452^c^	382^b^
SEM	0.53	1.41	1.75	1.645	5.12	0.24	0.09	6.09	10.97	5.84	9.37	7.91
*p* value	0.006	0.030	0.014	0.098	0.015	0.001	0.074	0.001	0.001	0.001	0.001	0.001
Regression analysis												
Linear	0.012	0.100	0.030	0.673	0.029	< 0.001	0.781	< 0.001	0.001	0.001	0.001	< 0.001
Quadratic	0.533	0.717	0.613	0.651	0.633	0.643	0.608	0.673	0.514	0.498	0.477	0.616
	80–83 weeks of age											
0%	52.67	51.23	103.90	102.81	291	14.55	4.70	374	675	353	571	488
10%	56.70	50.21	106.90	112.93	299	15.06	4.80	387	704	371	597	506
20%	55.04	51.11	106.15	107.69	297	14.97	4.76	385	695	369	593	502
30%	52.63	51.10	103.73	102.99	290	14.59	4.67	375	682	365	578	490
40%	53.70	49.90	103.60	107.62	290	14.72	4.61	378	683	368	583	494
SEM	1.14	1.33	1.86	3.459	5.20	0.25	0.09	6.45	11.65	6.15	9.86	8.37
*p* value	0.086	0.925	0.599	0.241	0.599	0.515	0.598	0.521	0.438	0.285	0.366	0.513
Regression analysis												
Linear	0.593	0.657	0.508	0.509	0.508	0.872	0.275	0.864	0.875	0.226	0.887	0.866
Quadratic	0.154	0.781	0.249	0.480	0.252	0.262	0.247	0.268	0.179	0.192	0.165	0.279
	84–87 weeks of age											
0%	60.07	47.72^ab^	107.79	125.88	302	15.09	4.87	388	701	366	593	507
10%	59.56	48.62^a^	108.18	122.50	303	15.30	4.84	394	716	377	606	514
20%	59.21	45.75^ab^	104.96	129.42	294	15.07	4.62	387	700	372	597	505
30%	56.62	43.52^b^	100.13	130.10	280	14.57	4.35	375	681	365	577	489
40%	59.59	46.69^ab^	106.28	127.63	298	15.60	4.57	401	724	391	618	524
SEM	1.37	1.05	2.07	2.951	5.76	0.31	0.09	7.99	14.43	7.68	12.22	10.32
*p* value	0.428	0.021	0.074	0.413	0.068	0.240	0.102	0.258	0.293	0.142	0.226	0.215
Regression analysis												
Linear	0.372	0.054	0.119	0.538	0.114	0.775	0.101	0.804	0.781	0.148	0.614	0.766
Quadratic	0.353	0.234	0.236	0.760	0.233	0.263	0.219	0.276	0.349	0.321	0.365	0.249
	76–87 weeks of age											
0%	50.70	50.59	101.29	100.22	284	14.18	4.58	365	658	344	557	476
10%	52.10	51.22	103.32	101.72	289	14.48	4.67	372	677	357	574	486
20%	50.97	48.66	99.63	104.75	278	13.98	4.47	359	649	345	554	469
30%	49.24	49.30	98.55	99.88	276	13.79	4.46	355	645	345	547	463
40%	50.09	48.64	98.73	102.98	276	13.90	4.44	357	645	348	551	467
SEM	0.82	0.72	1.36	1.540	3.88	0.21	0.06	5.35	9.70	5.23	8.22	6.98
*p* value	0.190	0.057	0.104	0.179	0.083	0.165	0.065	0.164	0.110	0.410	0.182	0.159
Regression analysis												
Linear	0.555	0.053	0.125	0.347	0.123	0.262	0.056	0.262	0.262	0.847	0.368	0.266
Quadratic	0.883	0.871	0.931	0.450	0.942	0.966	0.889	0.970	0.832	0.852	0.808	0.975

*Note*: Means within each column with uncommon superscript letters differ significantly (*p* < 0.05).

Abbreviations; AM, before noon; Ava P, available phosphorus; Ca, calcium; CP, crude protein; Dig Lys, digestible lysine; Dig Met, digestible methionine; Dig SAAs, digestible Sulphur amino acids; Dig Thr, digestible threonine; FI, feed intake; ME, metabolizable energy; Na, sodium; PM, after noon; SEM, standard error of the means.

^1^Calculated using feed‐intake data multiple to diets analysed composition in Table 1, the values are means of six replicates of 12 hens.

^2^Percentage change of nutrients between the morning and evening diets.

The dietary treatments did not affect the FI ratio between the morning and evening meals. Although the morning meals FI ratio in the first period (Weeks 76–79) was considerably (14%) lower than the evening meals, this pattern reversed in the second (80–83 weeks of age) and third (84–87 weeks of age) periods. In these later periods, the evening meals FI ratio was 3% and 12% higher than the morning meals, respectively. Over the entire experimental period (76–87 weeks of age), the overall FI ratio was nearly identical between the morning and evening meals (the morning meal FI ratio being only 0.75% higher than the evening meal).

### 3.2 EP and Quality Traits

The effects of CNL of AM/PM‐FR on EP performance indices are presented in Table 3. In response to increasing CNL of AM/PM‐FR, there was a significant effect with a quadratic trend (*p* < 0.05) on EW, FCR and IMFC during the first, third and overall experimental periods. The findings indicated that birds fed AM/PM‐FR at 30% CNL exhibited the lowest FCR and the highest IMFC. Throughout the entire experimental period, the birds fed AM/PM‐FR at 30% CNL improved FCR and IMFC compared to the control group by 4.57% and 17.55%, respectively. EW was highest in the birds fed AM/PM‐FR at a 10% CNL. EP and EM were not significantly affected (*p* > 0.05) by CNL of AM/PM‐FR during the entire or any of the experimental periods.

**TABLE 3:** Effect of AM/PM‐FR on egg production performance of aged laying hens.^1^

Items	76–79 weeks	80–83 weeks	84–87 weeks	76–87 weeks
Treatments^2^	Hen‐day egg production (%)			
0%	77.33	76.41	76.63	76.79
10%	79.40	77.32	77.17	77.97
20%	79.23	77.25	80.58	79.02
30%	79.97	79.27	78.38	79.21
40%	75.71	79.11	77.45	77.42
SEM	2.07	2.11	1.82	1.71
*p* value	0.580	0.837	0.583	0.830
Regression analysis				
Linear	0.171	0.763	0.185	0.252
Quadratic	0.123	0.996	0.216	0.289
	Egg weight (g/egg)			
0%	64.95^ab^	65.77	65.89	65.54^ab^
10%	65.30^a^	65.83	67.28	66.14^a^
20%	64.49^ab^	65.57	66.44	65.50^ab^
30%	64.89^ab^	64.80	64.71	64.80^ab^
40%	62.99^b^	64.52	65.35	64.28^b^
SEM	0.48	0.54	0.67	0.44
*p* value	0.019	0.324	0.102	0.043
Regression analysis				
Linear	0.008	0.979	0.007	0.001
Quadratic	0.047	0.541	0.041	0.035
	Egg mass (g/hen/day)			
0%	50.23	50.25	50.49	50.33
10%	51.85	50.90	51.92	51.57
20%	51.10	50.65	53.54	51.76
30%	51.89	51.37	50.72	51.33
40%	47.69	51.04	50.61	49.77
SEM	1.41	1.27	1.31	1.10
*p* value	0.257	0.980	0.445	0.659
Regression analysis				
Linear	0.247	0.645	0.174	0.184
Quadratic	0.060	0.855	0.137	0.135
	Feed conversion ratio (g FI/g EM)			
0%	1.834^a^	2.068	2.134^a^	2.013^a^
10%	1.829^ab^	2.101	2.082^ab^	2.004^ab^
20%	1.719^b^	2.097	1.961^b^	1.925^ab^
30%	1.768^ab^	2.021	1.975^b^	1.921^b^
40%	1.810^ab^	2.031	2.101^ab^	1.984^ab^
SEM	0.04	0.05	0.06	0.03
*p* value	0.037	0.775	0.105	0.046
Regression analysis				
Linear	0.045	0.473	0.013	0.037
Quadratic	0.039	0.645	0.020	0.036
	Income minus feed cost (10R/hen)^3^			
0%	246^b^	170	149^b^	188^b^
10%	262^ab^	157	165^ab^	195^ab^
20%	302^a^	157	205^a^	221^a^
30%	296^a^	186	183^ab^	221^a^
40%	262^ab^	176	135^b^	191^ab^
SEM	15.28	21.50	15.61	12.76
*p* value	0.045	0.865	0.198	0.016
Regression analysis				
Linear	0.048	0.367	0.031	0.044
Quadratic	0.035	0.325	0.022	0.045

*Note*: Means within each column with uncommon superscript letters differ significantly (*p* < 0.05).

Abbreviation: SEM, standard error of the means.

^1^Data are means of 6 replicates of 10 hens each.

^2^Percentage change of nutrients between the morning and evening diets.

^3^Egg income was calculated by multiplying the cost of average egg cost during the experimental period by the total amount of egg mass laid in experimental period. The average egg price was 16860(10R)/kg. Feed costs were calculated by multiplying the cost of each diet by the total amount of feed consumed in experimental period.

The effects of CNL of AM/PM‐FR on egg quality traits are presented in Table 4. All egg and shell quality traits exhibited a non‐significant response (*p* > 0.05) to CNL of AM/PM‐FR, except, in response to the increased CNL of AM/PM‐FR, the percentage of fractured eggs exhibited a significant (*p *< 0.025) and linear decrease at 87 weeks of age and approached significance (*p *< 0.089) at 79 weeks of age.

**TABLE 4:** Effect of AM/PM‐FR on egg quality of aged laying hen.s^1^

Items	Yolk weight (g)	Albumen weight (g)	Shell weight (g)	Haugh unit	Albumen index	Fractured egg (%)	ST (µm)	ESG (g cm^−3^)
Treatments^2^	Measured at 79 weeks of age							
0%	19.61	42.85	5.58	86.78	10.33	1.57^a^	515	1.077
10%	18.61	43.12	5.51	85.36	9.61	1.19^ab^	527	1.071
20%	17.62	41.87	5.67	84.31	9.27	1.02^ab^	510	1.076
30%	18.92	43.31	5.61	86.51	10.03	0.63^b^	520	1.074
40%	17.66	40.99	5.82	87.80	10.66	0.78^b^	536	1.078
SEM	0.53	1.11	0.23	2.61	0.61	0.24	16.14	0.00
*p* value	0.063	0.554	0.904	0.896	0.521	0.019	0.818	0.122
Regression analysis								
Linear	0.055	0.696	0.414	0.419	0.130	0.089	0.695	0.114
Quadratic	0.412	0.553	0.673	0.368	0.100	0.565	0.597	0.544
	Measured at 83 weeks of age							
0%	18.83	43.23	5.71	84.84	9.82	1.27	582	1.075
10%	19.15	43.10	5.74	85.19	9.20	0.93	581	1.076
20%	18.54	42.77	5.59	84.88	9.69	0.43	558	1.074
30%	18.64	44.30	5.45	87.11	10.45	1.06	566	1.077
40%	18.71	41.53	5.19	83.73	9.71	0.60	575	1.075
SEM	0.40	0.88	0.14	1.74	0.51	0.33	10.52	0.00
*p* value	0.841	0.309	0.054	0.738	0.567	0.394	0.454	0.861
Regression analysis								
Linear	0.799	0.405	0.612	0.460	0.538	0.252	0.165	0.777
Quadratic	0.887	0.319	0.257	0.445	0.984	0.474	0.212	0.390
	Measured at 87 weeks of age							
0%	18.74	42.35	5.62	90.10	11.56	0.56^a^	600	1.077
10%	18.79	42.50	5.54	88.09	10.50	0.53^a^	560	1.074
20%	17.55	43.71	5.24	84.68	9.74	0.00^b^	558	1.074
30%	19.75	43.90	5.34	89.47	10.92	0.00^b^	576	1.076
40%	18.13	42.78	5.27	86.07	10.37	0.12^b^	573	1.080
SEM	0.54	1.00	0.19	1.57	0.58	0.18	14.43	0.00
*p* value	0.084	0.735	0.551	0.111	0.278	0.018	0.281	0.058
Regression analysis								
Linear	0.894	0.275	0.134	0.247	0.162	0.025	0.107	0.154
Quadratic	0.964	0.331	0.562	0.423	0.215	0.248	0.132	0.392

*Note*: Means within each column with uncommon superscript letters differ significantly (*p* < 0.05).

Abbreviations; ST; shell thickness; ESG: egg special gravity and SEM: Standard error of the means.

^1^The values are means of six replicates of 6 samples (36 egg for each treatment were measured for egg quality).

^2^Percentage change of nutrients between the morning and evening diets.

The effects of CNL of AM/PM‐FR on egg components are presented in Table 5. As the CNL of AM/PM‐FR increased, yolk CP concentrations rose linearly (*p *< 0.012). Other components in the albumen, yolk and whole egg showed a non‐significant response (*p* > 0.05) to the CNL of AM/PM‐FR.

**TABLE 5:** Effect of AM/PM‐FR on egg composition of aged laying hens measured at 87 weeks of age.^1^

	Yolk (%)			Albumen (%)		Whole egg (without shell) (%)		
Items	Solids	Ether extract	Crude protein	Solids	Crude protein	Solids	Ether extract	Crude protein
Treatments^2^								
0%	48.77	28.79	18.27^ab^	11.84	11.37	26.29	10.20	14.07
10%	49.59	27.51	17.23^b^	11.81	10.86	26.21	10.15	13.28
20%	50.85	31.06	18.89^ab^	11.36	10.31	24.48	10.78	13.29
30%	50.47	30.08	18.85^ab^	11.04	10.77	25.81	10.96	13.72
40%	51.99	30.23	19.70^a^	11.53	10.78	25.76	10.31	13.90
SEM	1.54	0.79	0.48	0.37	0.48	1.17	0.44	0.41
*p* value	0.644	0.051	0.030	0.541	0.653	0.814	0.596	0.577
Regression analysis								
Linear	0.186	0.098	0.012	0.247	0.185	0.693	0.250	0.136
Quadratic	0.969	0.654	0.285	0.415	0.235	0.481	0.305	0.125

*Note*: Means within each column with uncommon superscript letters differ significantly (*p* < 0.05).

Abbreviation: SEM, Standard error of the means.

^1^The values are means of six replicates of two samples (12 egg for each treatment were measured for egg quality).

^2^Percentage change of nutrients between the morning and evening diets.

### 3.3 Blood Metabolites

The results for blood metabolite responses to the CNL of AM/PM‐FR are presented in Table 6. No significant differences were observed in blood metabolites and live functional enzymes between birds fed different treatments, except that the blood uric acid concentration increased linearly and significantly with increasing CNL of AM/PM‐FR; the blood uric acid concentration was highest in birds fed diets with a 30% CNL of AM/PM‐FR.

**TABLE 6:** Effect of AM/PM‐FR on blood metabolites and liver functional enzyme of aged laying hens measured at 87 weeks of age. ^1^

Items	Ca	P	Cr	UA	TP	Alb	ALP	ALT	AST
Treatments^2^	mg/dL			g/dL				U/L	
0%	16.6	6.4	0.5	4.9	6.5	2.2	537	11.0	238
10%	17.9	6.8	0.5	5.5	7.1	2.1	600	8.0	182
20%	16.7	6.1	0.3	5.7	7.0	2.5	709	8.0	219
30%	15.9	6.5	0.3	6.4	6.7	2.1	596	10.3	232
40%	17.9	6.2	0.5	6.0	7.0	2.1	619	9.3	140
SEM	0.74	0.48	0.06	0.40	0.37	0.22	119.52	1.42	32.50
*p* value	0.276	0.847	0.066	0.160	0.716	0.625	0.890	0.498	0.226
Regression analysis									
Linear	0.577	0.685	0.111	0.053	0.484	0.427	0.454	0.288	0.219
Quadratic	0.543	0.841	0.122	0.410	0.549	0.403	0.496	0.301	0.487

*Note*: Means within each column with uncommon superscript letters differ significantly (*p* < 0.05).

Abbreviation: Alb, albumin; ALP, alkaline phosphatase; ALT, alanine aminotransferase; AST, aspartate aminotransferase; Ca, calcium; Cr, creatinine; P, phosphorus; SEM, Standard error of the means; TP, total protein; UA, uric acid.

^1^The values are means of six samples/treatment.

^2^ Percentage change of nutrients between the morning and evening diets.

### 3.4 Tibia Bone Mechanical Properties and Mineral Contents

The effects of CNL of AM/PM‐FR on tibia bone characteristics, mineral concentration and strength are presented in Table 7. The dietary treatments did not significantly affect tibia bone mechanical properties and mineral concentration (*p* > 0.05).

**TABLE 7:** Effect of AM/PM‐FR on tibia bone characteristic, mineral concentration and strength of aged laying hens measured at 87 weeks of age.^1^

Items	Tibia bone characteristic				Tibia bone mineral concentration					Tibia bone strength		
	Weight	Ash	W/L	Ash/L	Ca	P	Mg	Mn	Zn	EB	FRSF	S
Treatments^2^	g	%	mg/cm		%			ppm		KJ	N	N/mm
0%	5.53	52.97	475	240	22.14	9.58	0.32	8	289	118	213	4.23
10%	5.43	51.73	475	236	22.02	9.63	0.31	7	325	116	179	3.57
20%	5.61	56.94	490	260	23.10	9.92	0.34	7	387	153	216	4.31
30%	5.37	52.51	477	233	22.35	9.61	0.32	8	365	115	178	3.55
40%	4.87	52.63	425	202	22.10	9.63	0.33	10	344	95	157	3.12
SEM	0.23	1.49	20.86	15.45	0.79	0.32	0.01	0.94	34.74	34.28	30.34	0.60
*p* value	0.244	0.166	0.272	0.181	0.864	0.941	0.690	0.404	0.368	0.401	0.654	0.657
Regression analysis												
Linear	0.299	0.276	0.132	0.107	0.453	0.568	0.588	0.148	0.096	0.455	0.253	0.252
Quadratic	0.156	0.268	0.084	0.072	0.457	0.571	0.783	0.132	0.145	0.401	0.654	0.657

Abbreviation: Ca, calcium; EB, energy between; FRSF, force that is required to reach the structural failure; L, length; Mg, Magnesium; Mn, Manganese; P, phosphorus; S, stiffness; SEM, Standard error of the means; W, Weight; Zn, Zinc.

^1^The values are means of six6 samples/treatment.

^2^Percentage change of nutrients between the morning and evening diets.

### 3.5 Apparent Nutrient Retention

The impact of CNL of AM/PM‐FR on TTANR is illustrated in Table 8. A significant linear response on EE and a quadratic response on Ca and phosphorus apparent retention were observed as the CNL of AM/PM‐FR increased. Laying hens fed AM/PM‐FR at a 20% CNL exhibited the highest nutrient retention, which was significantly greater than that of birds fed a control diet or AM/PM‐FR at 40% CNL.

**TABLE 8:** Effect of AM/PM‐FR on total tract apparent nutrient's retention in aged laying hens measured at 87 weeks of age.^1^

	Total tract apparent nutrient retention (%)					
Items	Dry mater	Ether extract	Crude protein	Ash	Calcium	Phosphorus
Treatments^2^						
0%	69.61	71.88^b^	66.02	44.90	40.45^b^	54.01^b^
10%	69.08	72.48^b^	66.81	50.73	48.44^a^	55.42^b^
20%	72.91	84.98^a^	71.07	50.45	48.35^a^	63.07^a^
30%	71.23	74.72^ab^	62.96	49.31	47.07^ab^	60.24^a^
40%	71.70	81.48^ab^	61.72	42.75	38.72^b^	37.37^c^
SEM	2.24	2.53	3.24	3.72	2.46	2.32
*p* value	0.746	0.008	0.326	0.474	0.033	< 0.001
Regression analysis						
Linear	0.355	0.049	0.227	0.083	0.012	0.001
Quadratic	0.663	0.394	0.179	0.064	0.009	< 0.001

*Note*: Means within each column with uncommon superscript letters differ significantly (*p* < 0.05).

Abbreviations; SEM: Standard error of the means.

^1^The values are means of 6 samples/treatment.

^2^Percentage change of nutrients between the morning and evening diets.

## 4 Discussion

### 4.1 Feed, Energy and Nutrients Consumption

The current experiment results indicated that laying hens offered AM/PM‐FR at 20% CNL had a 2%–3% lower consumption of feed, ME and Ca compared to birds on conventional feeding (control treatment) during the 76–79 weeks of age. The findings of this study demonstrate that by adjusting the diet's nutrient concentration in the morning and evening, hens can receive the right balance of nutrients when they need them most. This suggests that the AM/PM‐FR pattern adjusts the nutrient amounts proportionally to meet the demands of egg formation throughout the daily cycle. The findings of this study align with previous reports (El‐Razek et al. 2020; Jahan et al. 2024). The hypothesis is based on the understanding that egg albumen and shell are deposited over distinct periods (Jahan et al. 2024; Van Emous 2023; Yan et al. 2023). Consequently, hens may have higher protein and amino acid requirements in the morning and a greater Ca requirement in the afternoon.

### 4.2 EP and Quality Traits

The findings of the current study indicated that the productive (EW and FCR) and economic profit (IMFC) performance of aged laying hens improved through the use of AM/PM‐FR. These results are consistent with reports from several researchers (El‐Razek et al. 2020; Jahan et al. 2024; Lin et al. 2018; Molnár, Kempen, et al. 2018a, Molnár, Maertens, et al. 2018b; Van Emous 2023; Van Emous and Mens 2021; Zarghi et al. 2008). Egg protein synthesis necessitates an optimal composition of amino acids tailored to the hen's specific requirements. Notably, egg protein is recognized as an ideal amino acid mixture. The synthesis of egg protein in hens is a complex biological process that relies heavily on the availability of amino acids. These essential building blocks are sourced from the free amino acid pool, predominantly represented by the plasma pool. This pool is replenished through two primary mechanisms: intestinal absorption of dietary proteins and the catabolism of tissue proteins. To obtain the necessary amino acids from tissue catabolism, more tissue protein is needed for the formation of egg protein due to the limitation in sulphur amino acids (Hurwitz and Bornstein 1973). Given this dynamic, it becomes imperative to establish nutritional management that enhances the hen's efficiency. Such a strategy must consider both the biological availability of amino acids from various sources and the specific metabolic demands of the hen (Zarghi et al. 2008). Hens offered AM/PM‐FR diets at 30% CNL exhibited a 4.1% lower FCR compared to conventional feeding in the current study. These findings align with (El‐Razek et al. 2020; Jahan et al. 2024), which reported that FI in laying hens decreased and FCR improved when diets were implemented with the AM/PM feeding strategy.

Furthermore, another study indicated that feed efficiency was enhanced in hens receiving low protein and high Ca diets 8–10 h post‐oviposition, which aligns with the present findings (De los Mozos et al. 2014). However, in contrast to the current study's results, some other studies did not observe a significant impact of AM/PM feeding on productive performance (De los Mozos et al. 2014; Faruk et al. 2010). In line with the current study's findings, adjusting feeding timing for the necessary nutrients through AM/PM‐FR diets may contribute to improving the production rate (Van Emous 2023). This could support other studies (El‐Razek et al. 2020; Van Emous and Mens 2021) where hens under split feeding showed a tendency to significantly enhance EP compared to those on a traditional diet. Thus, the AM/PM feeding strategy may boost EP during the later phase of the production cycle and has the potential to improve the laying cycle, warranting further investigation (Jahan et al. 2024).

The current study showed, in response to the increased CNL of AM/PM‐FR, the percentage of broken eggs decreased linearly. Consistent with our findings, reported that providing laying hens with the majority of their daily Ca in the afternoon did not enhance shell quality compared to the control group receiving a diet with 3.5% Ca in both the morning and afternoon (Keshavarz 1998a, 1998b). The effect of dietary Ca level on eggshell formation depends on the total Ca intake during calcification (Leeson and Summers 2009).

In the current study in response to the increased CNL of AM/PM‐FR, the yolk CP concentration improved linearly. The AM/PM feeding strategy represents an innovative approach to poultry nutrition, aiming to enhance productivity and animal welfare in laying hen production systems for several reasons: (1) By adjusting the diet in the morning and evening, it can ensure that hens receive the right balance of nutrients when they need them most, potentially enhancing their overall health and EP (Molnár, Kempen, et al. 2018a, Molnár, Maertens, et al. 2018b). (2) This approach can lead to improved egg quality by meeting the hens' metabolic requirements at various stages of the day, which may positively influence shell strength, yolk colour and overall egg composition (Jahan et al. 2024). (3) Split feeding can help enhance feed efficiency by allowing producers to provide specific ingredients or supplements at optimal times, reducing waste and lowering feeding costs (Faruk et al. 2010). In the current experiment, there were no visible effects on other egg quality traits. Other studies (Londero et al. 2015; Van Emous 2023; van Emous and Mens 2021) also found no effects on egg quality when diets were divided into different parts of the day.

### 4.3 Tibia Bone Characteristic, Blood Metabolites and Liver Functional Enzyme

In the present study, although increasing the level of change in nutrient concentration in the morning and evening diets did not significantly affect tibia bone characteristics, mineral concentration and strength; blood metabolites; and liver functional enzymes, it was observed that in birds fed AM/PM‐FR at 20% CNL, indices such as tibia weight, ash content, weight‐to‐length ratio, ash‐to‐length ratio, mineral concentration and indices for assessing tibia bone strength reached their highest numerical values. In agreement with the current study, it is anticipated that providing higher dietary Ca levels in the afternoon may influence bone characteristics in laying hens, especially when the replenishment of Ca from the medullary bone becomes less accessible during egg formation (Cufadar et al. 2011; Zhao et al. 2020). The concentrations of blood serum Ca, phosphorus, total protein and alkaline phosphatase were unaffected by AM/PM‐FR, remaining within the normal physiological range. The concentration of liver functional enzymes in blood serum is typically regarded as an important indicator of overall health. The results of this research demonstrate that the implementation of the nutritional program does not adversely impact the body's health. This finding aligns with reports indicating that nutrient levels in afternoon diets do not need to match those in morning diets to maintain a consistent body weight (El‐Razek et al. 2020; Jahan et al. 2024). The increased uric acid concentration in the blood of birds, resulting from the elevated CNL of AM/PM‐FR, indicates that at the highest level of CNL between the morning and evening diets, the birds are compelled to draw from their reserves to meet the body's needs.

### 4.4 Apparent Nutrient Retention

The results of this study revealed that TTANR for EE, Ca, and phosphorus were highest in the birds that received AM/PM‐FR at a 20% CNL. The increased phosphorus requirement in the morning is essential to replace bone minerals that are reabsorbed overnight during shell formation (Clunies et al. 1992; Keshavarz 1998a, 1998b). Consequently, we hypothesized that it might be feasible to provide the hens with diets containing a sufficient amount of protein and amino acids only in the morning, and an adequate amount of Ca solely in the afternoon. This feeding strategy could help optimize FI, enhance nutrient absorption and potentially improve EP and quality. If successful, this approach has the potential to reduce the daily nutrient requirements of laying hens by increasing their utilization, thereby decreasing the excretion of Ca, phosphorus and N in the manure and helping to lower feed costs.

## 5 Conclusion

The results obtained from the current experiment suggest that, in the aged laying hens, by implementing split feeding at 30% CNLs between the morning and evening diets, it becomes feasible to improve FCR, economic efficiency and nutrient utilization via precision nutrition, while simultaneously enhancing egg quality.

## Author Contributions

H.Z. and M.T. designed and carried out the experimental trail. M.T. carried out the experimental trail lab analysis. H.Z. performed wrote the draft and reviewed the manuscript. A.J. performed the statistics. H.K. reviewed the manuscript.

## Funding

The authors have nothing to report.

## Disclosure

The authors declare that all of the authors listed on the manuscript employed at an academic or research institution where research or education is the primary function of the entity. Also, this manuscript is independently submitted by the authors.

## Ethics Statement

The authors confirm that the ethical policies of the journal, as noted in the journal's author guidelines page, have been adhered to and the appropriate ethical review committee approval has been received. The authors confirm that they have followed EU standards for the protection of animals used for scientific purposes and feed legislation.

## Conflicts of Interest

The authors declare no conflicts of interest.

## Data Availability

The data that support the findings of this study are available from the corresponding author upon reasonable request.
